# The “Great Debate” at Melanoma Bridge 2021, December 2nd–4th, 2021

**DOI:** 10.1186/s12967-022-03406-7

**Published:** 2022-05-10

**Authors:** Paolo A. Ascierto, Allison Betof Warner, Christian Blank, Corrado Caracò, Sandra Demaria, Jeffrey E. Gershenwald, Nikhil I. Khushalani, Georgina V. Long, Jason J. Luke, Janice M. Mehnert, Caroline Robert, Piotr Rutkowski, Hussein A. Tawbi, Iman Osman, Igor Puzanov

**Affiliations:** 1 Department of Melanoma, Cancer Immunotherapy and Innovative Therapy, Istituto Nazionale Tumori IRCCS “Fondazione G. Pascale”, Naples, Italy; 2 Melanoma Service, Memorial Sloan Kettering Cancer Center, New York, NY USA; 3 Netherlands Cancer Institute, Amsterdam, Netherlands; 4 Division of Surgery of Melanoma and Skin Cancer, Istituto Nazionale Tumori “Fondazione Pascale” IRCCS, Naples, Italy; 5 Department of Radiation Oncology, Department of Pathology and Laboratory Medicine, Weill Cornell Medicine, New York, NY USA; 6 Department of Surgical Oncology, Division of Surgery, The University of Texas MD Anderson Cancer Center, Houston, TX USA; 7 H. Lee Moffitt Cancer Center (MCC) & Research Institute, Tampa, FL USA; 8 Melanoma Institute Australia, The University of Sydney, Sydney, Australia; 9 Faculty of Medicine & Health, The University of Sydney, Sydney, Australia; 10 Charles Perkins Centre, The University of Sydney, Sydney, Australia; 11 Royal North Shore Hospital, Sydney, Australia; 12 University of Pittsburgh Medical Center, UPMC) Hillman Cancer Center, Pittsburgh, PA USA; 13 Department of Medicine, New York University Grossman School of Medicine, New York, NY USA; 14 Institut de Cancérologie Gustave Roussy Et Université Paris-Saclay, Villejuif, France; 15 Department of Soft Tissue/Bone Sarcoma and Melanoma, Maria Sklodowska-Curie National Research Institute of Oncology, Warsaw, Poland; 16 Melanoma Medical Oncology, Investigational Cancer Therapeutics, Division of Cancer Medicine, MD Anderson Brain Metastasis Clinic, UT MD Anderson Cancer Center, Houston, TX USA; 17 New York University Langone Medical Center, New York, NY USA; 18 Department of Medicine, Roswell Park Comprehensive Cancer Center, Buffalo, NY USA

**Keywords:** Melanoma; staging, Immunotherapy, Anti-PD-1, Anti-CTLA-4, Targeted therapy, BRAF inhibitor, MEK inhibitor, Adjuvant, Neoadjuvant, Surgery, Lymphadenectomy

## Abstract

The Great Debate session at the 2021 Melanoma Bridge virtual congress (December 2–4) featured counterpoint views from experts on seven important issues in melanoma. The debates considered the use of adoptive cell therapy versus use of bispecific antibodies, mitogen-activated protein kinase (MAPK) inhibitors versus immunotherapy in the adjuvant setting, whether the use of corticosteroids for the management of side effects have an impact on outcomes, the choice of programmed death (PD)-1 combination therapy with cytotoxic T-lymphocyte-associated antigen (CTLA)-4 or lymphocyte-activation gene (LAG)-3, whether radiation is needed for brain metastases, when lymphadenectomy should be integrated into the treatment plan and then the last debate, telemedicine versus face-to-face. As with previous Bridge congresses, the debates were assigned by meeting Chairs and positions taken by experts during the debates may not have necessarily reflected their respective personal view. Audiences voted both before and after each debate.

## Introduction

The Great Debate session at the 2021 Melanoma Bridge virtual congress (December 2–4) featured counterpoint views from experts on seven important issues in melanoma. The debates considered the use of adoptive cell therapy versus use of bispecific antibodies to treat melanoma, MAPK inhibitors versus immunotherapy in the adjuvant setting, whether the use of corticosteroids for the management of immune-related side effects has an impact on outcomes, the choice of cytotoxic T lymphocyte-associated antigen (CTLA)-4 or lymphocyte-activation gene (LAG)-3 inhibitor as the best option to combine with anti-programmed-death (PD)-1 therapy, whether radiation is needed for brain metastases, whether lymphadenectomy is best done before adjuvant therapy or later and telemedicine versus face-to-face visits. As with previous Bridge congresses, the debates were assigned by meeting Chairs and positions taken by experts during the debates may not have necessarily reflected their respective personal view. Audiences voted both before and after each debate.

## Adoptive cell therapy or bispecific antibodies in melanoma

### Nikhil I. Khushalani: in favour of adoptive cell therapy

Patients with advanced melanoma who progress after anti-PD-1 inhibitor therapy have limited treatment options. Studies of treatment with ipilimumab, alone or in combination with anti-PD-1 or high-dose interleukin (IL)-2, have reported response rates of around 25–30% in patients who failed on anti-PD-1 treatment, which represent the current benchmark for new treatment strategies in this population [1–4].

Adoptive cell transfer (ACT) with tumor-infiltrating lymphocytes (TIL) involves the infusion of autologous T cells obtained from the tumor microenvironment (TME) of the individual patient. These T cells are expanded and activated *ex-vivo* before reinfusion. Initial clinical trials of TIL therapy in melanoma occurred before the availability of immune checkpoint inhibitor therapy. For instance, a preliminary report in 20 patients with metastatic melanoma treated with TIL therapy and IL-2 after a single intravenous dose of cyclophosphamide as lymphodepletion resulted in response rates of 60% in IL-2 naïve patients and 40% in patients with prior IL-2 exposure [5]. Duration of response (DOR) was 2 to > 13 months. More recently, a meta-analysis identified 13 studies of TIL therapy and IL-2 following non-myeloablative chemotherapy in metastatic melanoma patients [6]. In 410 heavily pre-treated patients, including patients with brain metastases, the objective response rate (ORR) was 41% and the overall complete response rate was 12%. In patients who had received high-dose IL-2, the ORR was 43%, median overall survival (OS) was 17 months, and 1-year OS rate was 56.5%.

A recent phase II open-label multicenter study assessed the use of lifileucel, an autologous, centrally manufactured TIL therapy, in patients with advanced melanoma previously treated with checkpoint inhibitors and targeted agents [7]. A total of 66 patients received a nonmyeloablative lymphodepletion regimen, a single infusion of lifileucel, and up to six doses of high-dose IL-2. The ORR was 36% (3% complete responses) and disease control rate (DCR) was 80%. Median time to response was 1.4 months and one-year DOR was 69%. Overall, 92% of patients with a complete or partial response survived beyond one-year and only one-quarter of patients progressed after initial response to TIL therapy. In patients who were refractory to anti-PD-1 or PD-ligand (L)1 therapy, the ORR was 41% and DCR was 81%. Adverse events typically occurred early and declined over time. Another recent report in 10 therapy-naïve metastatic melanoma patients treated with lifileucel in combination with pembrolizumab reported an ORR of 60% and DCR of 90% with 4 of 6 responses ongoing after a median follow-up of 11.5 months [8].

There are no data to directly compare TIL therapy with the use of bispecific antibodies in cutaneous melanoma. However, in advanced uveal melanoma, TILs resulted in an ORR of 35% in 20 patients [9]. One patient had complete response of hepatic metastases, which was ongoing at 21 months after therapy. In comparison, treatment with tebentafusp, a bispecific antibody consisting of an affinity-enhanced T-cell receptor fused to an anti-CD3 effector, resulted in an ORR of 9% and a 6-month progression-free survival (PFS) rate of 31% in an open-label, phase III trial of 378 patients with previously untreated metastatic uveal melanoma [10].

TIL therapy is truly a form of precision medicine that involves a one-time procedure and administration. Reductions in the time required to generate the product have led to lower attrition rates. The use of lymphodepletion potentially addresses the immunosuppressive TME and may increase the bioavailability of cytokines. There is now an established track record of success of TIL in advanced cutaneous melanoma over three decades, with durable responses being reported even in PD-1 refractory patients. The toxicity profile is as expected in relation to lymphodepletion and high-dose-IL-2 and is manageable. Moreover, central manufacturing of the cellular product means the treatment is no longer restricted to specialized centers with cell therapy capability. In comparison, there are virtually no data with bispecific antibodies in melanoma refractory to immune checkpoint inhibitors, an area of great unmet need. The existing limited data indicate that bystander toxicity, including cytokine release syndrome (CRS) and neurotoxicity, may be a problem and thus require specialized expertise in cell therapy towards mitigation. In addition, multiple administrations are typically required and pharmacokinetic/pharmacodynamic properties must be considered. Finally, fixed dosing algorithms may lead to loss of flexibility in mitigating toxicity.

### Piotr Rutkowski: in favour of bispecific antibodies

Bispecific antibodies are engineered proteins composed of antigen-binding fragments from two different monoclonal antibodies. Advantages of bispecific antibodies include the large number of combination targets, developability, novel mode of action, high specificity and attractive economics. Interest in the use of bispecific antibodies to treat cancer has grown over the past two decades, with numerous trials now completed or ongoing.

Bispecific antibodies which redirect T cells to tumor cells are exemplified by bispecific T cell engagers (BiTEs), which consist of linked single-chain variable fragments of two different antibodies, one that binds to CD3 on T cells and one that targets a tumor antigen on tumor cells. After binding to the neoplastic cell, the BiTE antibody attracts T lymphocytes to this target cell, stimulating them to form adhesins and cytolytic substances such as granzyme, perforin and cytokines. The first BiTE antibody introduced into clinical practice was the anti-CD19 bispecific antibody, blinatumomab, which is currently indicated for the treatment of relapsed or refractory acute lymphoblastic leukemia (ALL). In a phase III trial with 376 patients with relapsed or refractory B-cell precursor ALL, blinatumomab resulted in significantly longer OS than chemotherapy [11].

Several anti-CD20 bispecific antibodies are also in development for lymphoma. Bispecific antibody constructs are also under investigation in solid tumors, although this has presented a challenge with obstacles including on-target, off-tumor toxicity, sparse T cell tumor infiltration and impaired TIL quality. In melanoma, dysfunctional infiltrating T cells are good targets for bispecific antibodies. Bispecific antibodies inhibited tumor growth in mice inoculated with B16 melanoma cells, resulting in the long-term survival of animals, while a second bispecific antibody that triggered the co-stimulatory CD28-molecule on the T-cell surface further increased tumor-cell killing in vitro and in vivo [12]. Bispecific anti-CD3 x anti-B7-H3 antibodies have also been shown to mediate T cell cytotoxicity to human melanoma in a xenograft mouse model [13]. Tumor-directed blockade of PD-L1 by a PD-L1 x CSPG4 bispecific antibody enhanced in vitro activation status, interferon (IFN)-γ production, and cytotoxicity of anticancer T cells and so may improve the efficacy of PD-1/PD-L1 checkpoint blockade for treatment of melanoma and other CSPG4-overexpressing malignancies [14].

Tebentafusp is a bispecific protein consisting of an affinity-enhanced T-cell receptor fused to an anti-CD3 effector that can redirect T cells to target glycoprotein 100-positive cells. In a trial of 378 patients with previously untreated metastatic uveal melanoma, treatment with tebentafusp significantly improved OS versus investigator's choice of single agent pembrolizumab, ipilimumab, or dacarbazine (73% versus 59%; p < 0.001) [10]. The most frequent adverse events with tebentafusp were cytokine-mediated events and skin-related events, including rash, pyrexia, and pruritus. These decreased in incidence and severity over time and resulted in treatment discontinuation in just 2% of patients.

**Fig. 1:**
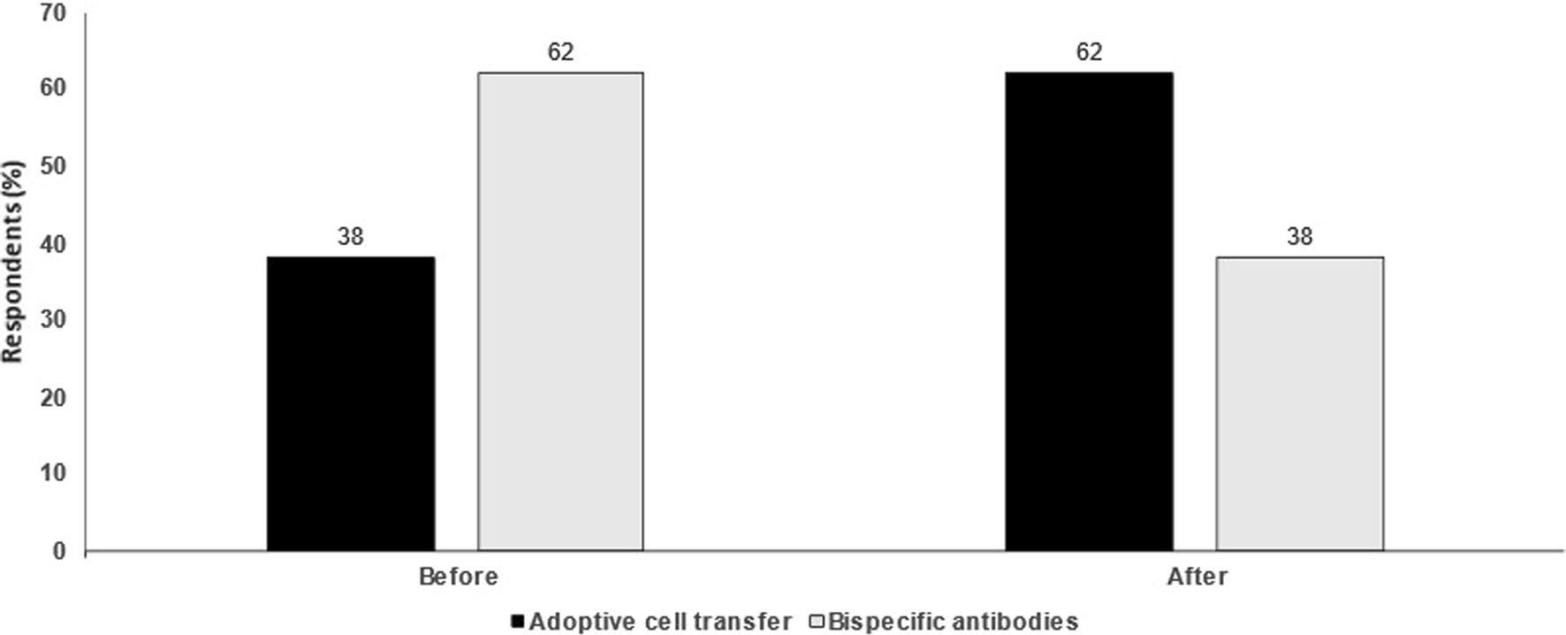
Adoptive cell therapy or bispecific antibodies in melanoma. Audience response before and after debate

Compared with ACT therapies, bispecific antibodies are available ‘off the shelf’ with no waiting time to treat a patient. In comparison, CAR T-cell manufacturing can take up to 3‑4 weeks. In terms of dosing, bispecific antibodies involve repeat dosing over time, while CAR T-cell therapy is provided as a single dose following lymphodepletion. CAR T-cell therapy may also be more immunogenic and associated with greater incidence of CRS (Fig. 1).

### Key points:

- TIL therapy involves a one-time administration and reductions in the time to generate the product have led to lower attrition rates, while central manufacturing means the treatment is no longer restricted to specialized centers.
- TIL therapy has an established track record of success in advanced cutaneous melanoma, with durable responses being reported even in PD-1 refractory patients.
- Advantages of bispecific antibodies include the large number of combination targets, developability, novel mode of action, high specificity, and attractive economics.
- Various classes of immunotherapeutic bispecific antibodies have different targets and may convert the TME from immunologically cold to hot and restore T cell function.
- While antibody-based therapeutics can only target proteins expressed on the cell surface, representing approximately 10% of the proteome, T-cell redirecting-based therapeutics can target any intracellular protein that is major histocompatibility complex (MHC) processed and presented.
- As compared to CAR T-cells, bispecific antibodies can be used ‘off-the-shelf’ with relatively simple production processes and less cytokine-release syndrome.

## MAPK inhibition versus immunotherapy in the adjuvant setting in melanoma

### Janice Mehnert: in favour of MAPK inhibition

The two pivotal adjuvant anti-PD-1 studies, the KEYNOTE-054 trial of pembrolizumab versus placebo in resected stage III melanoma and the CheckMate-238 trial of nivolumab versus ipilimumab in resected stage IIIB–IV melanoma, have both shown a clear and sustained recurrence-free survival (RFS) benefit in the anti-PD-1 treatment arm [15, 16]. This benefit was seen across all studied subgroups; in CheckMate-238, a benefit for nivolumab was observed with respect to RFS in nearly every subgroup tested, including those defined according to age, sex, disease stage, microscopic versus macroscopic nodal disease, ulceration status of the primary tumor, and BRAF status. Although these two studies slightly differed in their design, importantly all patients had prior complete lymph node dissection (CLND).

In the COMBI-AD trial, adjuvant therapy with the BRAF inhibitor dabrafenib in combination with the MEK inhibitor trametinib resulted in a significantly lower risk of recurrence in patients with stage III melanoma with BRAF V600E or V600K mutations compared with placebo [17]. The trial met its primary endpoint, with an estimated 3-year RFS rate of 58% in the combination group and 39% in the placebo group (hazard ratio [HR] for relapse or death, 0.47; P < 0.001). At 5-year follow-up, 12 months of adjuvant therapy with dabrafenib plus trametinib resulted in a longer duration of survival without relapse or distant metastasis than placebo [18]. The percentage of patients with distant metastasis-free survival (DMFS) was 65% with the combination and 54% with placebo and the HR for distant metastasis or death of 0.55 was similar to the 0.53 observed in KEYNOTE-054. In BRAF-positive stage III patients, MAPK-directed therapy has the most pronounced benefit in the first year, an OS benefit is noted, and there is evidence of increased relapse post-completion of therapy. In comparison, immunotherapy appears to result in less marked benefit in the first year but more sustained benefit over time. Also of note, the safety profile of dabrafenib plus trametinib was consistent with that observed in patients with metastatic melanoma with most toxic events reversible. However, treatment did result in 26% of patients having adverse events that led to permanent discontinuation of a trial drug, which may influence treatment choice.

In patients with stage II disease, the role of adjuvant therapy is less clear, with no clear benefit for most patients. In the KEYNOTE-716 phase III study of 976 patients with resected high-risk stage II melanoma, treatment with adjuvant pembrolizumab versus placebo showed a 35% reduction in the risk of recurrence [19]. Treatment also had a manageable safety profile, with grade ≥ 3 drug-related adverse events occurring in 16.1% of patients in the pembrolizumab group versus 4.3% with placebo; 15.3% discontinued due to a drug-related adverse event (2.5% with placebo). Immune-mediated adverse events were mostly mild-to-moderate in severity.

The BRIM-8 trial of adjuvant vemurafenib in resected, BRAF V600 mutation-positive stage IIC-III melanoma did not meets its primary endpoint of RFS in patients with stage IIIC disease [20]. As such, the trial failed to meet its criterion for analysis of the entire study. However, exploratory analysis of patients with stage IIC-IIIB disease did suggest a potential benefit in disease-free survival (DFS) in vemurafenib-treated patients. Prospective data and rigorous, clinically relevant biomarkers are needed to help identify patients with stage II disease who are most likely to benefit from adjuvant treatment.

Patient-centered considerations for therapeutic choice, which can include schedule and convenience of administration, side effect profiles, safety considerations, life circumstances (e.g., fertility), concomitant medical conditions and cost are also important. For example, rare cases of immunotherapy-induced type 1 diabetes have been reported, which can have a life-long impact on patients. This is an important consideration since for many patients, adjuvant therapy may not have been required to achieve cure – although it is beyond our capabilities right now to prospectively know who will or will not benefit from therapy. The incidence of other chronic immune-related adverse events may also be more frequent than previously recognized. As such, discussion of potential side effects with patients is critical in terms of therapeutic choice.

In conclusion, patients with the least amount of disease may be the best group to treat with BRAF-targeted therapy, which appears to be curative in the adjuvant setting even if not in the metastatic setting. MAPK targeted therapy is the only therapeutic approach to confer an OS benefit in adjuvant therapy trials to date. Finally, we do not know which patients will never have recurrent disease so, for these patients, the potentially permanent side effects of adjuvant therapy should be avoided if possible.

### Caroline Robert: in favour of immunotherapy

Three different treatment strategies are approved for adjuvant therapy in stage III melanoma based on the results of randomized, controlled trials; two of these are immunotherapies (pembrolizumab based on the KEYNOTE-054 trial and nivolumab based on the CheckMate-238 trial [15, 16]) and one is targeted therapy (dabrafenib in combination with trametinib) based on the COMBI-AD trial [17]). All three treatments have shown a benefit of treatment, with both anti-PD-1 therapy and immunotherapy reducing the risk of relapse in stage III patients.

In patients with BRAF-mutant melanoma, subgroup analysis of this population in KEYNOTE-054 at 3 years showed an HR for relapse or death of 0.51 for pembrolizumab versus placebo, the same as that reported with dabrafenib plus trametinib in COMBI-AD. However, the difference between active treatment and placebo was 25% with pembrolizumab and 20% with dabrafenib plus trametinib, and there was a trend for the curve versus placebo to become closer over time with targeted therapy but not anti-PD-1 therapy. Moreover, when the RFS curves for the individual trials are compared, the curve for dabrafenib plus trametinib appears to decrease steadily, whereas curves for immunotherapy drop more rapidly within the first 6 months, but more slowly thereafter, with the curves meeting at around 18 months [21].

In vitro, combined BRAF and MEK inhibition can induce rapid, non-mutational drug resistance, with a subpopulation of BRAF V600 mutant melanoma cells undergoing a reversible remodelling of mRNA translation [22]. These cancer persister cells tolerate targeted treatment and serve as a reservoir for acquired resistance and cancer relapse.

To date, there is no demonstration of a significant OS improvement with treatment in the adjuvant setting. Treatment with dabrafenib plus trametinib showed a numerical benefit in OS, but this did not reach the prescribed threshold for significance, so it is not yet possible to conclude a survival benefit for any treatment. DMFS has shown an improvement with both strategies but cannot be compared across trials due to different methodologies, with distant metastases occurring after local relapse included in KEYNOTE-054 but not COMBI-AD.

Safety considerations are also very important in choice of therapy. Most immune-related adverse events with pembrolizumab occurred during the first six months of treatment, some of which, e.g., endocrinopathies, can be persistent. However, certain events may be associated with better efficacy. Dabrafenib plus trametinib is associated with a higher rate of adverse events but these are not persistent or long-term, which is an advantage of targeted therapy. Neither immunotherapy nor targeted therapy has been shown to negatively impact patients’ quality of life compared with placebo.

One important question is what happens after relapse. However, data are limited and only from small, retrospective studies. In 147 patients who relapsed during or after adjuvant anti-PD-1 therapy (median time to relapse 4.6 months), response to targeted therapy was 78% if relapse occurred on anti-PD-1 and 90% if relapse was after anti-PD-1 [23]. Of those who recurred after adjuvant PD-1, two (40%) responded to PD-1 monotherapy, and two (40%) responded to ipilimumab-based therapy. In 85 patients after adjuvant targeted therapy (median time to relapse 17.7 months), response rates were 63% with anti-PD-1, 62% with ipilimumab plus nivolumab and 25% with targeted therapy rechallenge [24]. In terms of mode or relapse, there are no good data for comparison, but subjective experience may indicate more brain metastases after targeted than immunotherapy, although this requires investigation in a clinical trial.

**Fig. 2:**
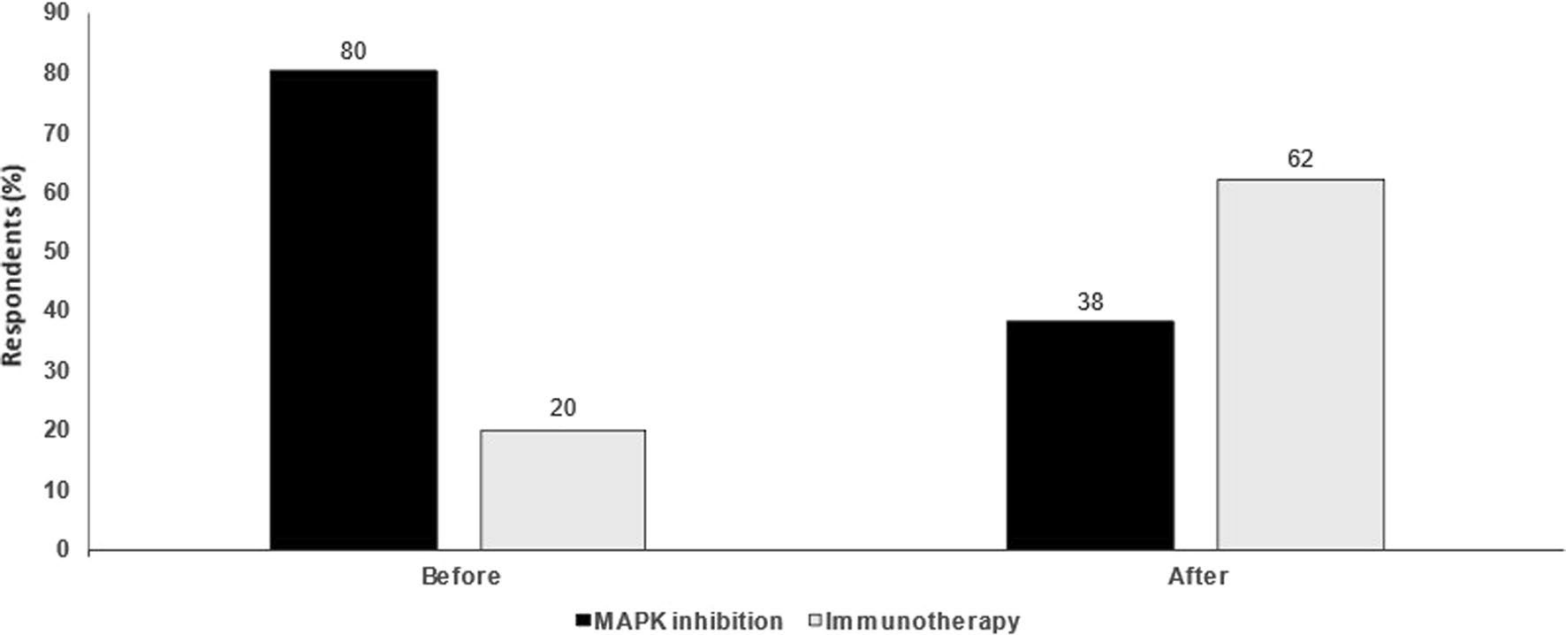
MAPK inhibition versus immunotherapy in the adjuvant setting in melanoma. Audience response before and after debate

In conclusion, the RFS benefit is similar for both immunotherapy and targeted therapy, but immunotherapy may offer a more durable benefit. There is evidence that targeted therapy, but not immunotherapy, may be a strong inducer of cancer cells that give rise to resistance. Conversely, in favour of targeted therapy, adverse events are more frequent but importantly are less persistent compared with immunotherapy. Mode of relapse and efficacy of second-line treatment after relapse are important issues which require more investigation. Finally, it is important to discuss the pros and cons of treatment options with each individual patient (Fig. 2).

### Key points:

- Adjuvant immunotherapy (pembrolizumab and nivolumab) and MAPK-targeted therapy (dabrafenib plus trametinib) have been shown to reduce the risk of relapse in stage III patients.
- MAPK-targeted therapy is the only therapeutic approach to show an OS benefit in adjuvant therapy trials to date, although this did not reach the prescribed threshold for significance.
- Immunotherapy appears to result in less marked benefit in the first year but more sustained benefit over time than targeted therapy.
- There is evidence that targeted therapy, but not immunotherapy, may be a strong inducer of cancer cells that give rise to resistance.
- Adverse events are more frequent but importantly are less persistent with targeted therapy compared with immunotherapy

## Could corticosteroids used for the management of side effects have an impact on the outcome of melanoma patients: Yes or No?

### Georgina V. Long: Yes

Several reviews have concluded that corticosteroids do not impact the efficacy of immunotherapy in patients with metastatic cancer. However, there is evidence to suggest that this conclusion is not correct because corticosteroids can have a detrimental impact on efficacy. In a pre-clinical study, dexamethasone decreased lymphocyte activation and proliferation during checkpoint blockade, impacting the ability of T cells to respond to cancer [25]. In humans, a pooled analysis of 576 patients treated with nivolumab reported an ORR of 31% [26]. This increased to 49% in patients with any selected (i.e., potentially immune-related) adverse event but decreased to 28% in patients with grade 3–4 selected adverse events. Moreover, in patients who received corticosteroids (for any adverse event), the response rate was 29%, suggesting that corticosteroids may have a detrimental impact on efficacy. In a retrospective analysis of 947 patients treated with anti-PD-1 therapy, early high-dose prednisone use for immune-related adverse events was associated with worse PFS and OS [27]. A pooled analysis of the CheckMate-067 and -069 trials of ipilimumab plus nivolumab shows an altered long-term outcome for those with adverse events, suggesting again, a detrimental impact of corticosteroids; the PFS was initially better in patients who discontinued because of adverse events than those who did not discontinue, (response rates of 58% and 50%, respectively), but in the longer-term, no difference was observed [28]. In the adjuvant setting, the RFS benefit was less for pembrolizumab versus placebo in the subgroup of patients who had ≥ 30 days of systemic steroid use than in the group without steroid use or < 30 days of systemic steroid use. In other words, corticosteroids appeared to blunt an otherwise potentially excellent RFS benefit [29].

The use of corticosteroids at baseline before starting checkpoint inhibitor therapy can also have a negative effect. In the ABC trial of nivolumab alone or in combination with ipilimumab for patients with active melanoma brain metastases, intracranial response was 6% in the cohort that were permitted high doses of corticosteroids for their symptoms, compared with much higher rates in cohorts in which corticosteroids were not permitted [30]. Similar results were seen in the CheckMate-204 trial of combination nivolumab plus ipilimumab, in which the best ORR in symptomatic patients receiving corticosteroids was only 17%, compared with 54% in asymptomatic patients [31]. In a study of patients with melanoma and significant pre-existing autoimmune disease treated with anti-PD-1, the ORR was 33%, somewhat lower than is typically expected with anti-PD-1 monotherapy. When patients were compared based on immunosuppressant use at baseline, those on baseline immunosuppression had a significantly lower response rate (15% versus 44% in patients not receiving baseline immunosuppressants) [32].

This data suggests we need to treat immune-related adverse events with more specific therapies, rather than with corticosteroids which blunt so many compartments of the immune system. Other drugs used to treat autoimmune disease may be repurposed to better manage immune-related adverse events in cancer. For example, in a retrospective analysis of patients with melanoma receiving immune checkpoint inhibitors, the IL-6 inhibitor tocilizumab was well-tolerated and an effective steroid-sparing treatment for the prevention and management of immune-related adverse events [33].

### Christian Blank: No

The first question is whether immune-related toxicity is predictive of response to immunotherapy, and there is considerable evidence to suggest this is the case. In a retrospective review of 298 patients with melanoma treated with ipilimumab, 85% experienced an immune-related adverse event [34]. Neither OS nor time to treatment failure (TTF) were significantly affected by the occurrence of immune-related adverse events; however, there was a trend to improved survival in the group who experienced toxicity. A pooled analysis of four nivolumab studies with 576 patients reported that ORR was significantly higher in patients who experienced treatment-related immunological adverse events of any grade compared with those who did not [26]. Similarly, in the adjuvant setting, the occurrence of immune-related adverse events was associated with improved RFS in patients treated with pembrolizumab after CLND of cutaneous metastatic melanoma [29]. Evidence is also provided by prospectively collected data from the Dutch Melanoma Treatment Registry of 1250 patients with advanced melanoma treated with first-line checkpoint-inhibition, of whom 312 (25%) had a grade ≥ 3 toxicity [35]. Patients with severe immune-related adverse events had a significantly improved median OS compared with patients without severe toxicity (23 versus 15 months).

Whether immunosuppression affects response is less clear. In the study of ipilimumab by Hovert et al. [34], neither OS nor TTF were significantly affected by use of systemic corticosteroids. Similarly the pooled analysis of four nivolumab studies did not show any effect of systemic immunosuppressant use on ORR [26]. However, Dutch Melanoma Treatment Registry data indicated survival was significantly decreased in patients who received tumor necrosis factor (TNF) inhibitors ± steroids for steroid-refractory toxicity compared with patients who received no immunosuppressant or steroids only (median OS of 17 versus 33 months) [35]. This association remained after adjusting for age, sex, WHO performance status, number of comorbidities, stage of disease, total number of metastases, lactate dehydrogenase (LDH), and immune checkpoint inhibitor regimen used. Moreover, immune checkpoint inhibitor therapy was reintroduced as often in patients who received anti-TNF as in patients who did not. The worse survival in anti-TNF-treated patients was also not linked to colitis. Melanoma-specific survival (MSS) was also shorter in patients who received TNF inhibition.

In contrast to this observation, it has been reported that mice that received anti-TNF added to combined CTLA-4 and PD-1-blockade had a higher rate of tumor control and survival than mice that were treated with CTLA-4- and PD-1-blockade only [36]. However, timing of anti-TNF is different in our cohort and most of our anti-TNF-treated patients also received high-dose steroids, which was not the case in the murine studies.

To date, the effect of TNF inhibition on tumorigenesis is unclear and may vary in different situations Further investiagtion is needed to confirm whetherr there is any detrimental effects of TNF inhibition. However, alternative stratgies may also need to be considered. Vedolizumab is a humanized monoclonal antibody used to treat ulcerative colitis, which acts by binding to α4β7 integrin on T lymphocytes, thereby reducing lymphocyte trafficking to the gut. In a retrospective study of 184 patients with cancer and immune-mediated diarrhea and colitis (IMDC), treatment with vedolizumab led to a similar IMDC response rate as infliximab treatment [37]. However. vedolizumab was associated with shorter duration of steroid use, lower IMDC recurrence, and more favorable OS than infliximab. Duration of treatments of immune-related adverse events may have been important, with patients who received ≥ 3 doses infliximab or vedolizumab having better OS compared to patients with 1–2 doses.

**Fig. 3:**
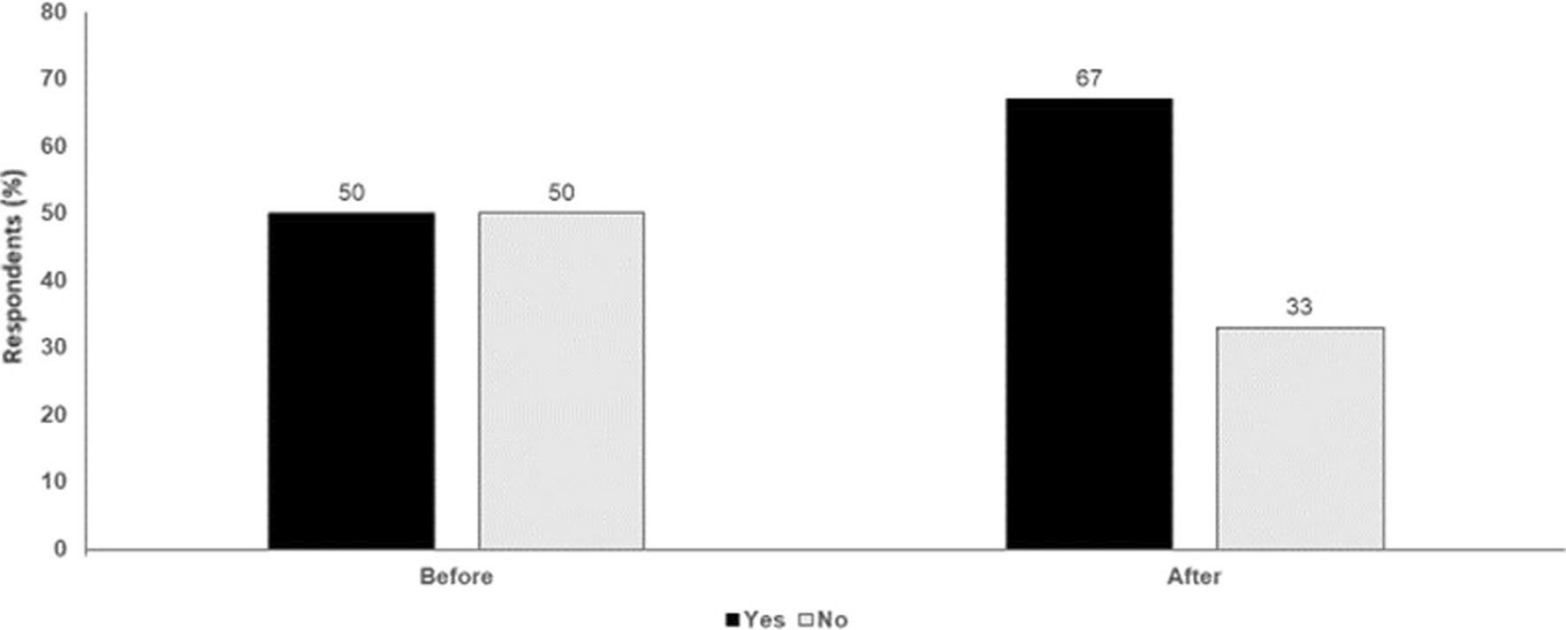
Could corticosteroids used for the management of side effects have an impact on the outcome of melanoma patients: Yes or No? Audience response before and after debate

In conclusion, corticosteroids used for the management of immune-related side effects do not appear to have an impact on the outcome of melanoma patients. However, the use of TNF inhibitors should be carefully monitored (Fig. 3).

### Key points:

- Corticosteroids may have a detrimental impact on efficacy of immune checkpoint inhibitors, although this remains unclear.
- As corticosteroids may blunt many compartments of the immune system, the use of more specific therapies aimed at specific mechanisms of an overactive immune system could be used to manage immune-related adverse events.
- Other drugs used to treat autoimmune disease may be repurposed to better manage immune-related adverse events in cancer, e.g., tocilizumab.
- The use of TNF inhibitors (with or without) steroids for steroid-refractory toxicity may worsen survival and so their use should be carefully monitored.

## PD-1 in combination with CTLA-4 or LAG-3: which one for which patient?

### Jason J. Luke: in favour of CTLA-4

The availability of various new treatment regimens for metastatic melanoma means choice of optimal first-line therapy has become more complex. However. the combination of nivolumab and ipilimumab have robust and mature survival data. In the phase III CheckMate-067 trial, nivolumab plus ipilimumab showed durable improved survival versus ipilimumab alone in patients with advanced melanoma at a median follow-up of 6.5 years, which represents the longest follow-up from a phase III melanoma trial in the modern era [38]. It is important to note that the greatest benefit of nivolumab plus ipilimumab is seen in high-risk patients with BRAF-mutant or PD-L1 negative tumors, and this has become accepted standard therapy over recent years.

The mechanisms of action of nivolumab and ipilimumab are not entirely clear but it appears as though nivolumab acts primarily in the TME whereas ipilimumab may in part act by priming the lymph nodes and the generation of de novo immune responses against the tumour. Clearly, data for anti-PD-1 plus the anti-LAG-3, relatlimab, are also promising, with significantly improved PFS for relatlimab with nivolumab versus nivolumab alone from the RELATIVITY-047 phase III trial of 714 patients with advanced melanoma [39]. However, these PFS data with a relatively short follow-up are the only data on the combination that we have to date. LAG-3 is a secondary dysfunction checkpoint on dysfunctional T cells, emphasizing that this combination is likely to be active in the TME of patients who have an active immune response at baseline.

Another important consideration is the activity of anti-PD-1 plus ipilimumab in patients who are refractory to anti-PD-1 monotherapy. In the KEYNOTE-006 trial, patients who received ipilimumab as first subsequent systemic therapy after disease progression on pembrolizumab had an ORR of 15.5% [40]. Similarly, a prospective trial of ipilimumab plus pembrolizumab following progression on anti-PD-1 immunotherapy reported an ORR of 29% [41]. Interestingly, most responses occurred in patients with intermediate to non-T-cell-inflamed tumors at baseline, supporting the idea that CTLA-4 blockade may enhance the development of de novo antitumor responses that may benefit patients who do not have PD-1-high or inflamed tumors at baseline.

A disadvantage of nivolumab plus ipilimumab is the toxicity compared to anti-PD-1 monotherapy. In the 6.5-year follow-up of CheckMate-067, grade 3/4 treatment-related adverse events were reported in 59% of nivolumab plus ipilimumab patients compared with 24% of patients receiving nivolumab monotherapy [38]. However, this toxicity can be mitigated in clinical practice by using the ‘flip-dose’ regimen of nivolumab 3 mg/kg plus ipilimumab 1 mg/kg (N3I1) rather than nivolumab 1 mg/kg plus ipilimumab 3 mg/kg (N1I3). In the CheckMate-511 trial, survival outcomes for the two regimens were essentially identical [41]. However, the N3I1 regimen demonstrated an improved safety profile compared with N1I3, with grade 3/4 treatment-related adverse events in 19% versus 34% of patients. Other studies have also suggested that low-dose ipilimumab with anti-PD-1 maintains efficacy while decreasing toxicity [42].

Another consideration is the concept of treatment-free survival. In CheckMate-067, median treatment-free interval was 27.6 months for nivolumab plus ipilimumab compared with 2.3 months for nivolumab alone [38]. Similarly, patients alive and treatment-free at 6.5 years dramatically favored the combination, indicating that even though a significant proportion of patients may discontinue treatment due to adverse events early on, many of these patients have favorable outcomes without further therapy. Whether this might be true of anti-PD-1 plus relatlimab is unknown.

In conclusion, the PD-1/CTLA-4 combination has the most robust and mature data, is most active in high-risk patients and may offer long-term benefit with treatment-free survival. Moreover, the N3I1 dose regimen mitigates toxicity without impacting efficacy and should be the preferred backbone for future triplet combinations, e.g., with LAG-3, TIGIT, oncolytic viruses, toll-like receptor (TLR) agonists, targeted therapies, and radiation.

### Paolo A. Ascierto: in favour of LAG-3

The CheckMate-067 trial of nivolumab plus ipilimumab in patients with advanced melanoma has reported long-term durable survival versus ipilimumab alone [38]. In patients who responded to the combination, tumor regression tended to be more rapid and deeper than responses seen with nivolumab monotherapy. However, not all patients responded, with the combination tending to show most benefit in patients with BRAF-mutant disease, elevated LDH or M1c stage melanoma. Given the higher toxicity of the combination, treatment decisions are complex and multifactorial, a key aspect of which is identifying patients most likely to benefit. However, the role of predictive biomarkers is still to be defined.

New emerging pathways for future combination with anti-PD-1/PD-L1 compounds include anti-LAG-3 agents, such as relatlimab. LAG-3 is an immune checkpoint that inhibits the activation of its host cell, thereby contributing to a more immunosuppressive TME and contributing to anti-PD-1 resistance. Relatlimab plus nivolumab demonstrated clinical activity and a manageable safety profile in a phase I/II trial in heavily pretreated patients with melanoma and progression on prior anti-PD- 1/anti-PD-L1 therapy [43]. In the phase III RELATIVITY-047 trial in previously untreated patients, relatlimib plus nivolumab demonstrated superior PFS benefit versus nivolumab alone at a median follow-up 13.2 months (10.1 versus 4.6 months, HR for progression or death, 0.75; p = 0.006) [39]. This is comparable to the HR of 0.76 reported in the CheckMate-067 trial, although median follow-up in that trial was longer at 20.7 months.

The PFS benefit associated with relatlimib plus nivolumab was observed regardless of LAG-3 expression status (< or ≥ 1%), with HRs for both groups similar to that for the overall intent-to-treat population. These findings suggest that LAG-3 expression alone does not predict benefit from relatlimib plus nivolumab, although there was a trend toward improved median PFS among LAG-3 expressors in both arms. The combination of relatlimib plus nivolumab also reduced the risk of progression after next line of systemic therapy, per investigator assessment, or death (i.e., PFS-2) versus nivolumab alone.

Data are also provided from the neoadjuvant setting. In the PRADO trial of patients with Response Evaluation Criteria in Solid Tumors (RECIST) 1.1 measurable stage III melanoma, two cycles of neoadjuvant ipilimumab plus nivolumab resulted in a pathological response rate of 71%, with 50% of patients having a pathological complete response (pCR) and 61% a major pathologic response (MPR) [44]. Similarly, a study of patients with clinical stage III or oligometastatic stage IV melanoma with RECIST 1.1 measurable, surgically resectable disease that assessed relatlimab combined with nivolumab reported a 59% pCR rate and 66% MPR rate [45]. Patients with MPR had improved RFS versus those without. Thus, relatlimab plus nivolumab appears at least as effective as nivolumab plus ipilimumab as neoadjuvant treatment.

With regard to safety, 59% of patients receiving ipilimumab plus nivolumab had a grade 3/4 treatment-related adverse event and 42% had a treatment-related adverse event that led to treatment discontinuation in CheckMate-067 [38]. In comparison, treatment-related adverse events that led to discontinuation of trial therapy occurred in 14.6% of patients treated with relatlimab plus nivolumab in RELATIVITY-047 (versus 6.7% with nivolumab monotherapy) [39]. Treatment-related adverse events associated with relatlimab and nivolumab were manageable and reflected the safety profile typically seen with immune checkpoint inhibitors. Although treatment with these two agents led to an increase in the incidence of grade 3/4 treatment-related adverse events, these events occurred at a lower rate than has been observed with other immune checkpoint inhibitor combinations. Thus, in terms of ‘doing no harm’ the relatlimab plus nivolumab combination may have an advantage over nivolumab plus ipilimumab.

**Fig. 4:**
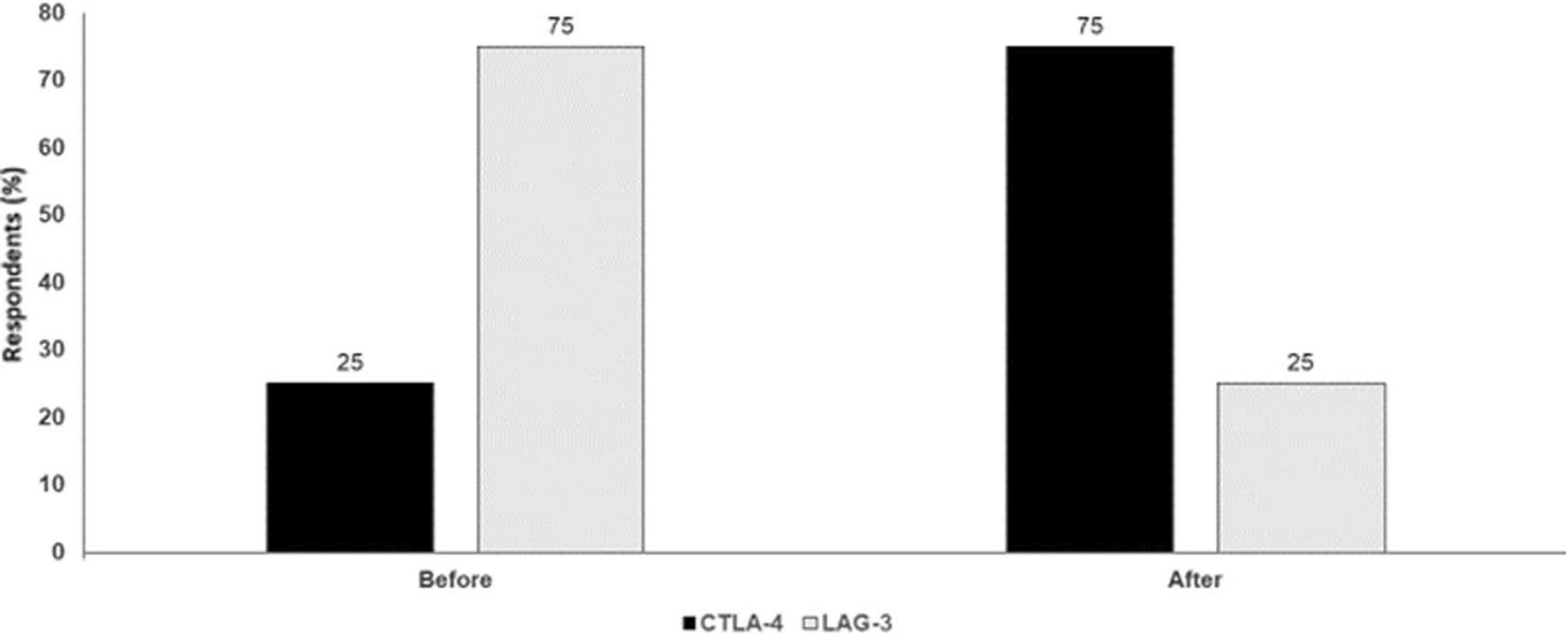
PD-1 in combination with CTLA-4 or LAG-3: which one for which patient? Audience response before and after debate

However, it is important to emphasise that the combination of anti CTLA-4 with anti PD-L-1 still represents an important treatment option, with ipilimumab extending the tail of the survival curve longer-term. One further option going forward may be triplet combinations. These are being investigated in the phase I/II CA224-048 study in which relatlimab will be administered in combination with nivolumab and either ipilimumab or an indolamine-2,3-dioxygenase (IDO)-1 inhibitor in patients with advanced malignant tumors (Fig. 4).

### Key points:

- Anti-PD-1 and anti-CTLA-4 in combination has the most robust and mature data, is most active in high-risk patients and may offer long-term benefit with treatment-free survival.
- The ‘flip-dose’ regimen of nivolumab 3 mg/kg plus ipilimumab 1 mg/kg mitigates toxicity without impacting efficacy and should be the preferred backbone for future triplet combinations.
- Data for anti-PD-1 plus anti-LAG-3 are promising, but to date are less robust, being PFS data with a relatively short follow-up.

## Brain metastases: do you need radiation? Yes or No

### Sandra Demaria: Yes

In the CheckMate 204 trial of nivolumab plus ipilimumab in patients with melanoma brain metastases, treatment was effective in asymptomatic patients but less so in patients with symptomatic disease [46]. Among patients with symptoms who did respond, disease control was durable, suggesting that interventions designed to relieve these patients of their symptoms or reduce steroid therapy use might improve their responsiveness, with possible strategies including stereotactic radiotherapy (SRT).

In contrast to combination therapy, the efficacy of single-agent ipilimumab or anti-PD-1 has been disappointing and inferior to results seen with radiation [47, 48]. However, a retrospective review of patients who underwent definitive stereotactic radiosurgery (SRS) for melanoma brain metastases showed that median survival in patients who also received ipilimumab was 21.3 months, versus 4.9 months in patients who did not receive ipilimumab [49]. The 2-year survival rate was 47.2% with SRS plus ipilimumab versus 19.7% with SRS alone. Another retrospective study also showed that combined ipilimumab and SRS was associated with favorable locoregional control and improved survival, and that concurrent delivery was the most effective strategy [50]. However, central nervous system toxicity requiring steroids was more frequent in patients receiving SRS during ipilimumab and the combination may also cause a temporary increase in tumor size, possibly associated with an increased immunomodulatory effect. Concurrent pembrolizumab with brain SRS also appears to be effective at reducing the size of brain metastases in patients with metastatic melanoma [51].

Radiation to the brain metastases in combination with immune checkpoint blockade can potentially improve not only local but also systemic metastasis control, mediated via the activation of anti-tumor T cells. Radiation has effects that can promote both, the priming and effector phase of the anti-tumor immune response [52]. Radiation induces the release of antigens during cancer cell death coupled with proinflammatory signals that trigger dendritic cells to uptake and cross-present tumor antigens to T cells. In addition, radiation can enhance the infiltration of activated T cells into the tumor and increase the expression of MHC class I by the cancer cells [53]. Importantly, the radiation doses and fractionation used are different in whole brain radiation therapy (WBRT), SRS and SRT, thus different approaches to treatment of brain metastases are likely to have a different impact on the immune system [54]. Preclinical work in peripheral tumors suggests that hypofractionated doses, such as used in SRT, are more effective at activation of systemic anti-tumor immunity in combination with immune checkpoint inhibitors [55]. Evidence of an abscopal effect after radiation to the brain is provided by a study of 21 patients with disease progression after ipilimumab [56]. Of these, 13 underwent radiation for melanoma brain metastases, with seven experiencing a tumor response outside of the radiation field at a median of one month after radiotherapy.

In conclusion, radiation provides local control of brain metastases, and can also induce or strengthen anti-tumor immune responses in combination with immune checkpoint inhibitors by improving the ability of T cells to infiltrate the tumor and recognize the cancer cells. However, serious side effects can include cognitive impairment (with WBRT) and radionecrosis (with SRS).

### Hussein A. Tawbi: No

Radiation clearly has a role in the treatment of brain metastases, but the more relevant question is whether radiation should be used before systemic therapy. Around 90% of patients with brain metastases present with concomitant systemic disease that also requires therapy. Responses with systemic agents occur in more than 50% of patients, can occur rapidly and can be highly durable. Moreover, shrinkage of intracranial tumors should mean subsequent radiation approaches are more effective and less toxic.

The targeted combination of dabrafenib plus trametinib has shown clinical activity in patients with melanoma brain metastases, with rapid responses in up to 60% of patients, but the median duration of response is relatively short [57]. A retrospective analysis of encorafenib plus binimetinib also reported intracranial activity in patients with BRAF-mutant melanoma brain metastases, including patients previously treated with targeted therapy [58]. With immunotherapy, intracranial response rates are lower than with targeted therapy, but responses are durable and associated with extracranial response [48].

In the CheckMate 204 trial, nivolumab plus ipilimumab was associated with a high intracranial response rate (57%) in patients with asymptomatic melanoma brain metastases [Ta46]. Among asymptomatic responders, responses were rapid (median 1.6 months) and 87% were ongoing at 3-year follow-up. In comparison, patients with symptomatic melanoma brain metastases had an ORR of 17%. Three-year intracranial PFS was 54%, with similar rates for extracranial and global disease, and OS was 72% in asymptomatic patients, versus 19% and 37% in the symptomatic cohort. In landmark OS analysis, 92% of asymptomatic patients with a response at week 12 were alive at 3 years.

For patients who are symptomatic, there may be a role for vascular endothelial growth factor (VEGF) inhibition, such as pembrolizumab in combination with lenvatinib or bevacizumab-based combinations. However, there may also be a role for cytoreduction, via surgery, targeted therapy, or SRS. In a retrospective study, early surgical resection before immunotherapy improved survival in patients without previous immune checkpoint blockade [59]. Triplet combinations of targeted and immunotherapy may also have value in patients that are symptomatic or on steroids. For example, the TriDENT study of nivolumab plus dabrafenib plus trametinib in patients with PD-1 naïve or refractory BRAF-mutated metastatic melanoma has reported promising clinical activity in patients with brain metastases [60]. Other triplets, such as encorafenib plus binimetinib plus nivolumab and vemurafenib plus cobimetinib plus atezolizumab are also being investigated.

Other novel combinations that may have lower toxicity than ipilimumab plus nivolumab may also be an option. In addition to low dose ipilimumab plus pembrolizumab, nivolumab plus relatlimab appears to have lower toxicity than nivolumab alone and a PFS comparable with ipilimumab plus nivolumab. A phase II trial of nivolumab plus relatlimab in patients with untreated melanoma brain metastases with a primary endpoint of 12-week ORR is underway.

There does appear to be a benefit from immunotherapy plus SRT with the potential of an abscopal effect. However, all the evidence to support this is from retrospective studies and there is a need for prospective data. Moreover, radiation necrosis is a significant problem, and its occurrence appears to be higher with immunotherapy. Predictors of radiation necrosis risk include dose of radiation and the number and size of lesions; given that systemic agents can reduce the number and size of lesions, this may result in less radiation being needed and hence less toxicity.

Nivolumab combined with ipilimumab has high activity and durability in asymptomatic melanoma brain metastases and may be considered for upfront therapy with SRS, as is being investigated in the ABC-X trial. However, treating all lesions upfront has the risk of overtreatment and may involve having to treat more and larger lesions than necessary, meaning the same risk of radiation necrosis. In comparison, waiting for salvage therapy may miss the window of opportunity during which time lesions are smaller and so may require whole brain radiation therapy rather than SRS. An alternative approach, being used by the MD Anderson Brain Metastasis Clinic, involves systemic therapy upfront with early evaluation at 8 weeks and SRS for patients with non-responding lesions.

**Fig. 5:**
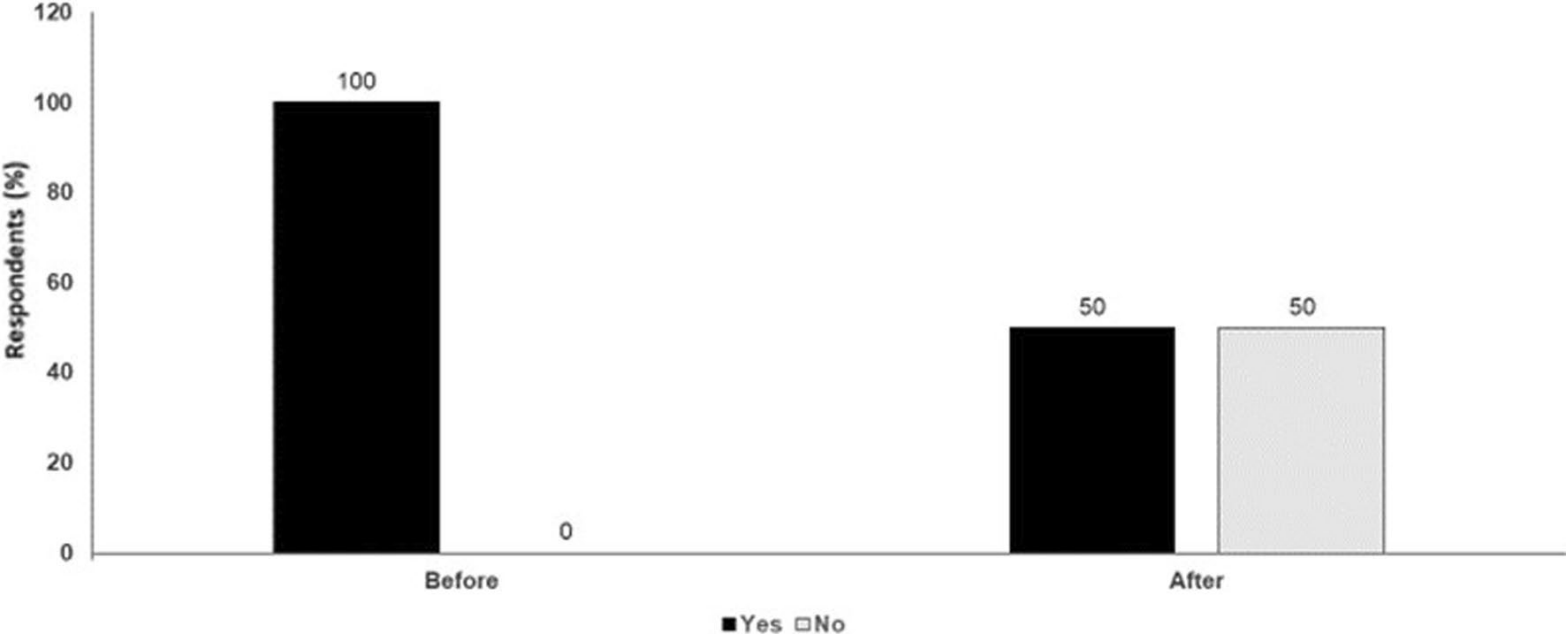
Brain metastases: do you need radiation: Yes or No? Audience response before and after debate

In conclusion, targeted and immunotherapies can be safe and effective for patients with melanoma brain metastases, with high rates of durable responses with upfront combination immunotherapy. Incorporation of SRS may have a role but optimal sequencing remains unclear and needs to be investigated in prospective randomized trials. Finally, novel treatments are needed to increase efficacy and decrease toxicity (Fig. 5).

### Key points:

- Systemic therapy with targeted therapy and immunotherapy has a high rate of intracranial responses. Combination immunotherapy induces rapid and durable responses in asymptomatic patients and should be considered for upfront therapy.
- Potential synergy between radiation and immunotherapy has been consistently reported in retrospective studies, with hypofractionation of radiation more likely to result in such synergy.
- There is some evidence that radiation applied to brain metastases of melanoma can enhance systemic responses to immune checkpoint blockade.
- Radiation has a critical role and is a standard of care, especially stereotactic radiosurgery.
- Choice of radiation dose, schedule and field should take into consideration the associated toxicities.
- Judicious use of radiation, potentially after systemic agents, in a multi-disciplinary setting could optimize the benefit while limiting radiation toxicities.

## Lymphadenectomy: a before or later approach?

### Corrado Caracò: in favour of before

The first question is whether it is correct to perform a treatment in all patients when 80% of them will not benefit? The answer to this is no. In data from the National Cancer Institute of Naples, Italy, only 19% of patients with a positive sentinel lymph node (SLN) had further node-positivity after complete lymph node dissection (CLND). Likewise, adjuvant treatment will only benefit around 20% of patients. In the future, biomarkers may give a better risk stratification and new indications for treatment with better identification of patients most likely to benefit.

Another question is does performing lymphadenectomy before or later (i.e., to treat micrometastatic disease or clinical nodal disease) result in similar outcomes? Historically, immediate node dissection according to the WHO Melanoma Program had no impact on survival compared with regional node dissection delayed until the appearance of regional-node metastases. Node dissection offered increased survival in patients with node metastases only [61]. In patients with intermediate thickness melanomas (1–4 mm), immediate lymph node dissection improved survival versus clinical observation of the lymph nodes [62]. Predictors of outcome were tumor thickness, the presence of tumor ulceration, trunk site, and patient age (> 60 years). More recently, the Multicenter Selective Lymphadenectomy Trial (MSLT-1) reported that wide excision and SLN biopsy with immediate lymphadenectomy for nodal metastases detected by SLN biopsy improved survival outcomes compared with wide excision and nodal observation, with lymphadenectomy for nodal relapse [63]. In another study of positive-SLN patients, nodal RFS was improved in patients who underwent CLND compared to those without CLND [64]. In the MSLT-2 trial, which randomized patients with sentinel-node metastases to immediate CLND or nodal observation with ultrasonography, immediate CLND was associated with improved survival without nodal recurrence compared to delayed radical dissection only after relapse in patients with pathologically detected metastases [65].

There may be a prognostic role of non-sentinel nodes. Immediate CLND with additional positive non-sentinel nodes was associated with improved MSS compared with salvage CLND in analysis of patients with a positive SLN biopsy at a single centre between 1991 and 2013 [66]. In MLST-2, the cumulative rate of non-sentinel node metastasis was higher among patients in the observation group who had nodal recurrence than in patients in the dissection group who had positive findings on pathological assessment or nodal recurrence [65]. Non-sentinel node metastases, identified in 11.5% of the patients in the dissection group, were a strong, independent prognostic factor for recurrence.

The next question is whether adjuvant treatment can be used to treat non-sentinel node residual disease. In adjuvant trials of anti-PD-1 therapy and targeted therapy, all patients were resected [15–17]. However, could the benefits of adjuvant treatment observed in these trials in part be lost in the absence of CLND? In a retrospective cohort of SLN-positive patients managed with nodal surveillance, adjuvant treatment did not alter patterns of initial recurrence. There was the same relative risk of nodal recurrence in patients with or without adjuvant therapy, despite a high SLN basin nodal recurrence rate [67]. Thicker primary melanomas, ulceration, larger SLN tumor deposit, and higher disease stage were all factors associated with recurrence.

Another question is whether immunotherapy is as effective for the treatment of nodal disease. In the KEYNOTE-054 trial of adjuvant pembrolizumab for resected stage III melanoma, there was only a 4%-point reduction in loco-regional recurrence with treatment versus placebo (18% versus 14%) [15]. Similarly, in patients with high-risk stage IIB or IIC melanoma, skin or lymph node regional recurrence was 6.4% with pembrolizumab versus 8.4% with placebo [19].

Whether neoadjuvant treatment impacts outcomes also need to be addressed. Neoadjuvant trials have resulted in better outcomes than trials in the adjuvant setting, with 50–80% of patients achieving a pCR. It is possible that immunotherapy is more effective in patients with a higher tumor nodal burden than in patients with micrometastatic disease.

Patients that might benefit from CLND upfront include those with early recurrence or severe toxicity during adjuvant treatment, patients aged < 18 or > 80 years who were excluded from trials, and those with stage IIIA disease with a tumor load of < 1.0 mm.

### Jeffrey E. Gershenwald: in favour of later

The treatment goals of regional lymph node basin management include pathological staging, regional control, potential cure, and minimizing morbidity. The historical approach to the regional nodal basin following lymphatic mapping and sentinel lymph node biopsy, a standard of care for many years, has been early therapeutic CLND in SLN-positive patients, with the rationale being to identify and remove non-SLN metastases. These can be prognostically significant and can influence staging, thereby contributing to clinical decision-making. This approach can improve regional control, reducing in-basin failure/loss of regional control, and may also have a beneficial impact on survival, as suggested by the MSLT-1 study [63]. However, overall, less than 20% of patients have tumor-involved non-sentinel lymph nodes at CLND. These patients have similar predictors of non-sentinel lymph node involvement and survival, with a high risk of both regional failure and distant recurrent disease.

Whether CLND is necessary in SLN-positive patients was addressed in two landmark clinical trials. In the MLST-2 study, immediate CLND improved disease-free survival (DFS) and nodal RFS compared with observation in patients with melanoma and SLN metastases [65]. However, the rate of lymphedema was four-fold higher in patients with CLND. Moreover, distant recurrence was the most frequent type of recurrence and was similar in both groups. Recurrence in the nodal basin, as the sole site of recurrence, was observed in 7.7% of patients in the observation group, as compared with 1.3% in the dissection group; this difference is only 6.4%. Overall, there was no difference in MSS between groups, and no real signal of a survival benefit in any patient subgroup, including those with the highest tumor burden. This trial was the basis for a paradigm shift in treatment, alongside the smaller DeCOG-SLT phase III trial which randomized 483 patients with cutaneous melanoma and a positive SLN to CLND or observation [68]. At 5-year follow-up, no significant differences were seen between the groups in DMFS, RFS or OS. As in MSLT-2, tumor burden did not affect survival outcomes, with no differences between groups with tumor load ≤ 1 mm or > 1 mm. For patients with a positive SLN, these two randomized clinical trials support only a limited role for early CLND.

Indeed, the most recent National Comprehensive Cancer Network (NCCN) clinical guidelines suggest surveillance is generally preferred [69]. However, exceptions noted include patients with a preference for surgery due to the logistical burden of surveillance, when primary tumor and SLN tumor burden suggest a higher likelihood of additional region involvement (although this also associated with distant metastases), or when adjuvant therapy is not pursued.

Meticulous surgical technique for accurate nodal staging is required in the ‘post-CLND for all sentinel lymph node-positive patients’ era. There is a theoretical argument that lack of additional non-SLN involvement from the CLND procedure could potentially impact clinical decision-making regarding adjuvant therapy, since some patients might be upstaged because of the additional nodal involvement identified at the time of + CLND. It is known that MSS is very heterogeneous across the four American Joint Committee on Cancer (AJCC)-8 stage III subgroups [70, 71] most oncologists will offer adjuvant therapy for patients with stage IIIB or higher disease. Patients with stage IIIA disease as determined by SLN biopsy information only may not be offered adjuvant therapy due to an overall favorable MSS profile. Despite this concern, it is likely that exceedingly few patients would be upstaged from AJCC-8 IIIA to IIIB (i.e., T1a/T1b/T2a primary melanoma and at least four positive lymph nodes), supporting that CLND results would have little overall impact on the decision to offer adjuvant therapy for these patients.

In a retrospective study of over 6000 SLN biopsy patients treated across Australia, Europe, and US between 2017 and 2019, 1154 were positive and had initial negative distant staging [72]. Of these, 84% had active surveillance and only 16% underwent CLND. Around 40% had adjuvant therapy, which was primarily anti-PD-1 treatment. In patients who received adjuvant treatment without undergoing prior CLND, all isolated nodal recurrences were resectable. In multivariate analysis, CLND improved isolated nodal RFS but not all‐site RFS. These initial real‐world outcomes align with randomized trial findings, including in those receiving adjuvant therapy.

**Fig. 6:**
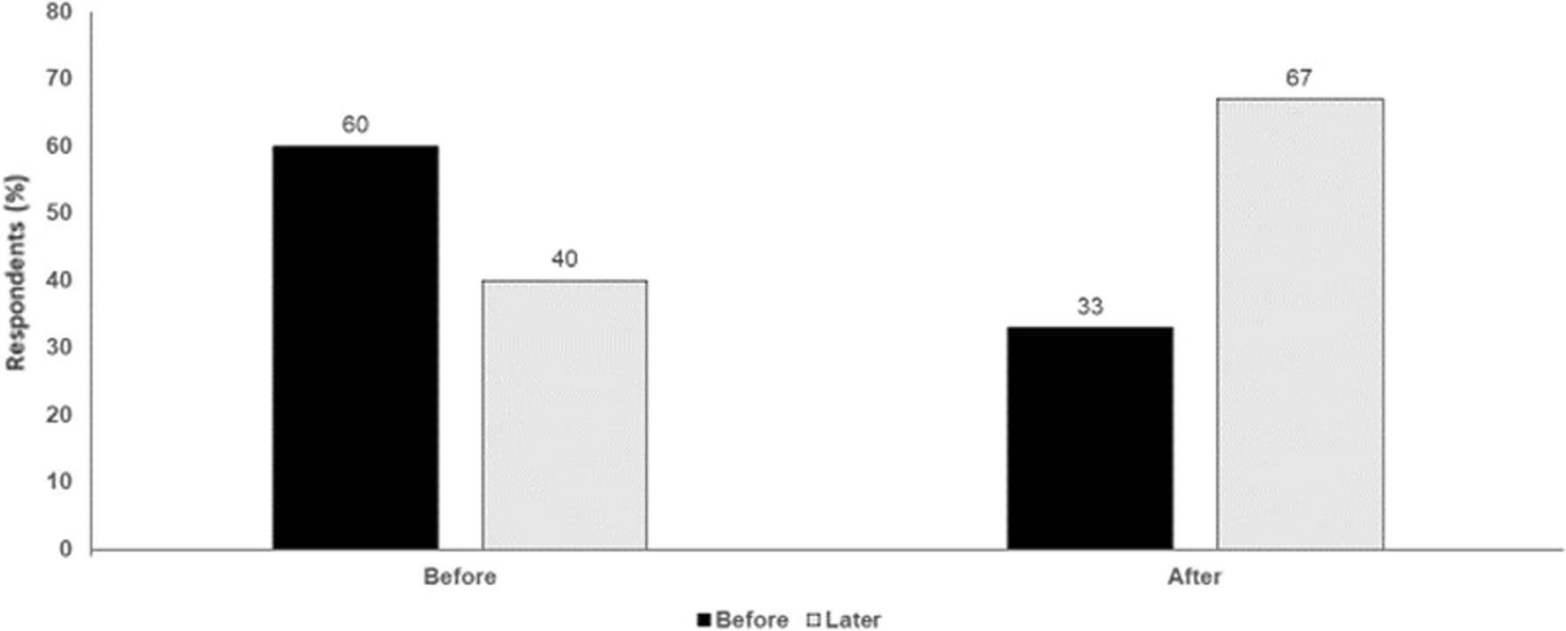
Lymphadenectomy: a before or after approach? Audience response before and after debate

For patients with clinically detected regional lymph nodes, wide excision of primary tumor and therapeutic lymph node dissection has been the standard recommendation in previous NCCN guidelines. However, the v1.2022 update now promotes the option to consider neoadjuvant therapy, preferably in the context of a clinical trial [69]. This is based on evidence from several recent clinical trials. In an early trial, treatment with neoadjuvant ipilimumab plus nivolumab was associated with a higher pCR rate and improved RFS versus nivolumab monotherapy [45]. However, the trial was stopped early due to concerns about progression and very high toxicity. Different dosing regimens have largely addressed the toxicity issue and a pooled analysis of six neoadjuvant immunotherapy or targeted therapy clinical trials reported that 40% of patients had a pCR, which was correlated with improved RFS and OS [73]. In patients receiving immunotherapy with pCR, near pCR or partial pathological response, very few relapses were seen with a 2-year RFS rate of 96%. Recently, neoadjuvant relatlimab plus nivolumab reported a 59% pCR rate and 66% MPR rate in patients with clinical stage III-IV melanoma [45]. In the PRADO trial, an extension cohort of the phase II OpACIN-neo study, patients that achieved pCR or near-pCR in the index lymph node did not undergo therapeutic lymph node dissection, which was associated with reduced surgical morbidity [44]. These data suggest the future possibility of omitting of an informed and individualized approach to omitting CLND (Fig. 6).

### Key points:

- The management of microscopic nodal disease remain controversial due to the inefficacy of adjuvant treatment to reduce positive sentinel node basin nodal recurrence.
- Neoadjuvant approaches seem to be effective with about 60% of pathological complete responses, but further analyses are necessary.
- Based on the results of randomized clinical trials, there has been a very significant shift in the management of the SLN-positive regional node basin, from near routine CLND to active surveillance and nodal observation for the vast majority of patients.
- The role, timing, and extent of lymphadenectomy for patients with regional node metastasis continues to evolve.

## Telemedicine versus face-to-face visits: which is better?

### Allison Betof: in favour of telemedicine

Telemedicine as an addition to our usual practice is already widely adopted as a standard of care and can offer several benefits. Patient-centered considerations, including improving the patient experience, patient access to care, and limiting financial toxicity, are all important and may benefit through increased adoption of telehealth. Aspects of telemedicine may also offer benefits to physicians.

To date, there are limited data on the patient experience of telehealth. However, a large US survey of patient experience in routine radiation oncology practice, that spanned pre-COVID-19 and the COVID-19 pandemic, found high patient satisfaction [74]. No significant differences were seen in patient satisfaction between office and telemedicine consultations, including the appointment experience versus expectation, quality of physician's explanation, and level of physician concern and friendliness. Although more patients considered office visits preferable to telemedicine when surveyed at the beginning (first visit) and end (last visit), the proportion of patients who reported that telemedicine was better than or no different to an office visit increased, suggesting physicians may have been getting better at telehealth consultations, and/or that patients became more comfortable with the process. Importantly, telemedicine in this survey was mostly done by telephone only, with no formal telemedicine platform—the development of such systems should lead to even better experiences for patients. In addition, there was a trend towards patients reporting telemedicine was better regarding the amount of time spent with physician and more patients considered telemedicine better in terms of treatment-related costs (i.e., lost wages, travel expenses). Those patients who preferred in-person office consultations were those with better performance status and those who were married/partnered, both groups which may have had fewer difficulties with access to face-to-face care.

In another survey, which included patients with sarcoma at a UK hospital during COVID-19, patient satisfaction with telemedicine was high [75]. Most patients were happy to receive test/imaging results by telehealth. A concern for many physicians is communicating bad news by telemedicine; however, the proportion of patients who reported they would not want to hear bad news by telemedicine was less than half (48%) of those surveyed. Although, if given an either/or choice, face-to-face was preferred by more patients, similar numbers of patients were happy for consultations to be mostly telemedicine or mostly face-to-face.

Patient access is another important consideration. Telemedicine can help overcome travel barriers by enabling consultations from remote and/or rural locations and reduces the caregiver burden in patients with poor overall health who may need assistance. Clinical trial access can also be improved; although participation will require office visits, aspects such as electronic consent, some toxicity assessments, and survivorship checks van be done through telehealth.

The financial costs of clinic visits can also be a burden for patients. These include the costs of travel (i.e., public transport, hospital parking), costs of associated childcare, and lost wages due to missed work. In 2019 in the US, the national economic burden for patients, including out-of-pocket and time costs, associated with cancer care was projected to be $21.1 billion [76]. A survey by the US Veterans Health Administration reported significant financial and time savings with high satisfaction with telemedicine for elderly patients with cancer [77]. Interestingly, a significant environmental benefit associated with reduced travel was also noted.

In addition to benefits for patients, physicians can also benefit through the increased adoption of telemedicine. Multidisciplinary and multi-institutional collaboration is facilitated, as is the involvement of caregivers who may be able to dial-in from other (i.e., work-based) locations. Physician workload appears to be similar, if not less.

Telemedicine is here to stay so the important question is how it can be best implemented. Clinician-reported barriers to effective telemedicine have been the use of telephone rather than video, lack of private space for appointments, and lack of nursing presence [75]. Improved video platforms, with integration of translation services, dedicated clinic space, and nursing involvement can help address these concerns. Improved ease of telemedicine licensure across state lines, which has been a major problem in the US, also needs to be addressed.

### Janice Mehnert: in favour of face-to-face visits

Although here to stay, there are many situations in cancer care for which telemedicine should not be relied upon. The obvious benefits of telemedicine for some patients, namely the reduced financial cost, easier scheduling, and improved convenience, need to be balanced against the more accurate assessments and compassionate communication provided by real-life, in-person consultations.

The ease with which we can use telemedicine in oncology depends on part on the nature of the visit. For example, pre-surgical evaluations, new radiation oncology evaluations and, in medical oncology, new, treatment/change of treatment, and end-of-life evaluations, may not be suited to telemedicine. Telemedicine can miss subtle physical examination findings (e.g., murmurs, crackles, evolving skin lesions). It also obviates the ability to get real-time diagnostic assessments (laboratory assessments, x-rays, imaging) and to offer acute treatments (e.g., Intravenous steroids, pain medications). Telemedicine can also generate the need for a follow-up visit when assessments performed are suboptimal.

In telemedicine, symptomatology dictates care and treatment decisions are made based largely on patient complaints. Objective findings are limited and there is the potential for diagnostic accuracy to be skewed. Evaluation of an insurance claims database with over four million patients and 69 million claims showed that care episodes initiated via telemedicine more frequently generated related visits within a 30-day period, suggesting the initial consultation may have been suboptimal [78]. Patients with cancer need to attend in-person for treatment and many offices are equipped for ‘one-stop’ laboratory assessments and scans. Although patients in remote settings may benefit, for patients not in these settings, attendance in person may be just as convenient.

Telemedicine also misses the emotional connection between doctors and patients. In-person visits are “more than a pat on the back” with patients deriving emotional support from the treatment team, something which is lost with telemedicine. In addition, telemedicine apps may not be easy for certain populations of patients to use, and some, in particular the elderly, may find the technology intimidating and anxiety-inducing.

Telemedicine is a new and evolving field with quality metrics yet to be determined. At present, its future is uncertain. In the US, payers in multiple states are still developing policies, which are subject to change, and are not necessarily supportive of telemedicine. It is also often unclear to what extent, if at all, telemedicine visits will be reimbursed in the future. If reimbursed poorly, it is doubtful telemedicine will continue to any major degree. In the US, pan-state or country licensing is also a limiting factor.

Finally, ‘Zoom fatigue’ is a genuine issue, with exhaustion after lengthy screen time a documented phenomenon. Hospital administrators may book multiple visits in short sessions and very few practices employ medical staff support in telemedicine triage or documentation.

**Fig. 7:**
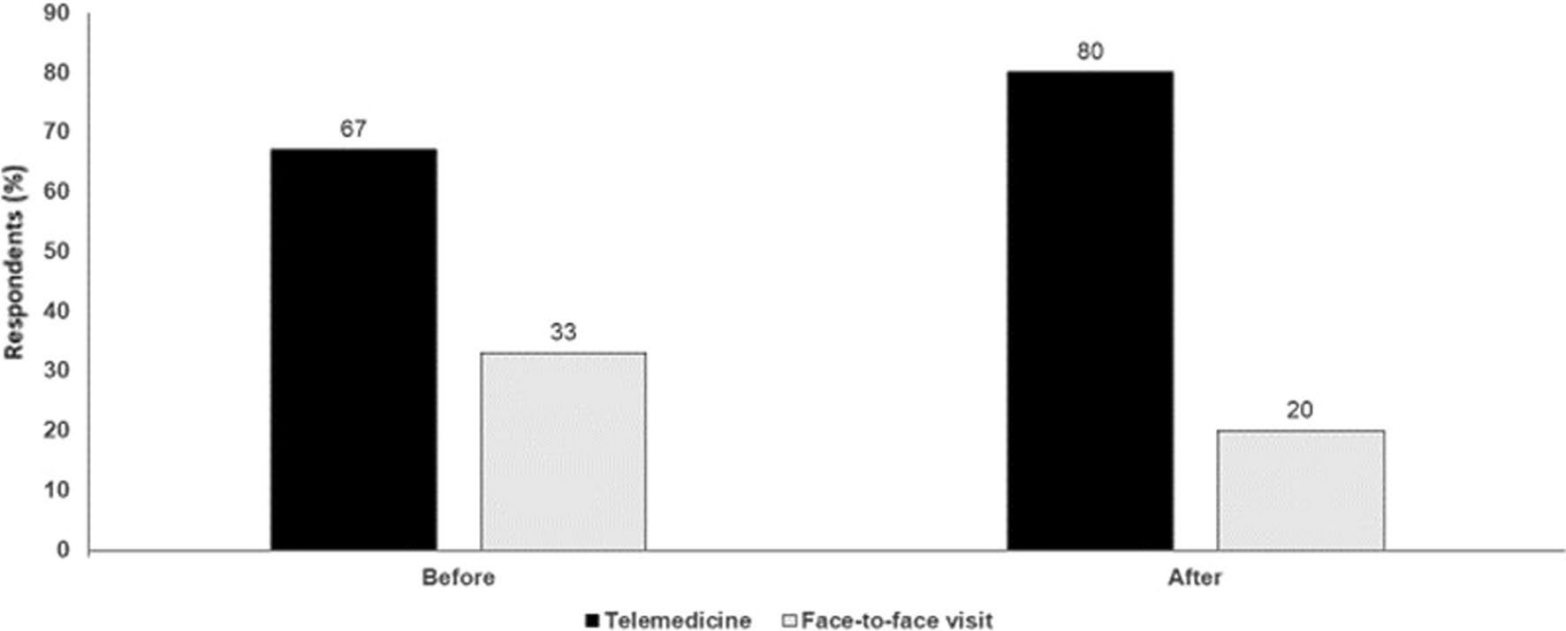
Telemedicine versus face-to-face visits: which is better? Audience response before and after debate

In conclusion, telemedicine can reduce diagnostic accuracy and convenience may not be cumulative if additional assessments are ultimately needed. Telemedicine can also reduce the emotional component of care that is so essential in medicine, especially in oncology. Questions about continued reimbursement also threaten its future (Fig. 7).

### Key points:

- Telemedicine can offer several benefits, including improved patient experience, patient access to care, and reduced financial toxicity,
- Aspects of telemedicine may also offer benefits to physicians, with increased multidisciplinary and multi-institutional collaboration and caregiver involvement.
- However, telemedicine can reduce diagnostic accuracy and convenience may not be cumulative if additional assessments are ultimately needed.
- Some patients may find the technology needed for telemedicine intimidating and anxiety-inducing.
- Telemedicine can also reduce the emotional component of care that is so essential in medicine, especially in oncology.

## Conclusions

Counterpoint views from leading experts on seven topical issues in melanoma management were debated during these sessions. Given the hybrid virtual/in-person format of the congress, presentations were not intended as a rigorous assessment of the field but rather provided opportunities to consider important areas of debate. It is hoped that these discussions can focus attention on these issues, stimulating further debate and encouraging the research needed to improve our understanding of different therapeutic approaches.

## Data Availability

Not applicable.

## Abbreviations

ACT: Adoptive cell transfer
AJCC: American Joint Committee on Cancer
ALL: Acute lymphoblastic leukemia
BiTE: Bispecific T cell engager
CLND: Complete lymph node dissection
CTLA-4: Cytotoxic T-lymphocyte-associated antigen-4
CRS: Cytokine release syndrome
DCR: Disease control rate
DFS: Disease-free survival
DMFS: Distant metastases-free survival
DOR: Duration of response
HR: Hazard ratio
IDO: Indoleamine 2,3-dioxygenase
IFN: Interferon
IL: Interleukin
IMDC: Immune-mediated diarrhea and colitis
LAG: Lymphocyte-activation gene
LDH: Lactate dehydrogenase
MAPK: Mitogen-activated protein kinase
MHC: Major histocompatibility complex
MPR: Major pathologic response
MSLT: Multicenter Selective Lymphadenectomy Trial
MSS: Melanoma-specific survival
NCCN: National Comprehensive Cancer Network
ORR: Objective response rate
OS: Overall survival
pCR: Pathological complete response
PD-1: Programmed death-1
PD-L1: Programmed death ligand-1
PFS: Progression-free survival
RECIST: Response Evaluation Criteria in Solid Tumors
RFS: Recurrence-free survival
SLN: Sentinel lymph node
SRS: Stereotactic radiosurgery
SRT: Stereotactic radiotherapy
TIL: Tumor-infiltrating lymphocyte
TLR: Toll-like receptor
TME: Tumor microenvironment
TNF: Tumor necrosis factor
TTF: Time to treatment failure
VGEF: Vascular endothelial growth factor
WBRT: Whole brain radiation therapy
